# Road traffic death coding quality in the WHO Mortality Database

**DOI:** 10.2471/BLT.23.289683

**Published:** 2023-08-22

**Authors:** Junjie Hua, Li Li, Peishan Ning, David C Schwebel, Jieyi He, Zhenzhen Rao, Peixia Cheng, Ruotong Li, Yanhong Fu, Jie Li, Wanhui Wang, Na Zhang, Guoqing Hu

**Affiliations:** a Department of Epidemiology, School of Public Health, Sun Yat-sen University, Guangzhou, China.; b Department of Epidemiology and Health Statistics, Xiangya School of Public Health, Central South University, 172 Tong Zi Po Street, Changsha, 410072, China.; c Department of Psychology, University of Alabama, Birmingham, United States of America.; d Department of Child, Adolescent and Women's Health, Capital Medical University, Beijing, China.

## Abstract

**Objective:**

To evaluate the precision and dependability of road traffic mortality data recorded in the World Health Organization Mortality Database and investigate how uncorrected data influence vital mortality statistics used in traffic safety programmes worldwide.

**Methods:**

We assessed country and territory-specific data quality from 2015 to 2020 by calculating the proportions of five types of nonspecific cause of death codes related to road traffic mortality. We compared age-adjusted road traffic mortality and changes in the average annual mortality rate before and after correcting the deaths with nonspecific codes. We generated road traffic mortality projections with both corrected and uncorrected codes, and redistributed the data using the proportionate method.

**Findings:**

We analysed data from 124 countries and territories with at least one year of mortality data from 2015 to 2020. The number of countries and territories reporting more than 20% of deaths with ill-defined or unknown cause was 2; countries reporting injury deaths with undetermined intent was 3; countries reporting unspecified unintentional injury deaths was 21; countries reporting unspecified transport crash deaths was 3; and countries reporting unspecified unintentional road traffic deaths was 30. After redistributing deaths with nonspecific codes, road traffic mortality changed by greater than 50% in 7% (5/73) to 18% (9/51) of countries and territories.

**Conclusion:**

Nonspecific codes led to inaccurate mortality estimates in many countries. We recommend that injury researchers and policy-makers acknowledge the potential pitfalls of relying on raw or uncorrected road traffic mortality data and instead use corrected data to ensure more accurate estimates when improving road traffic safety programmes.

## Résumé

**Objectif:**

Déterminer le niveau de précision et de fiabilité des informations concernant le nombre de décès liés aux accidents de la route enregistrées dans la base de données de l’Organisation mondiale de la Santé sur la mortalité, mais aussi étudier l’influence des données non corrigées sur les statistiques de mortalité, qui jouent un rôle essentiel dans les programmes de sécurité routière à travers le monde.

**Méthodes:**

Nous avons évalué la qualité des données propres à chaque pays et territoire entre 2015 et 2020 en calculant la proportion que représentent cinq types de codes non spécifiques relatifs aux causes de décès dans des accidents de la route. Nous avons comparé la mortalité routière selon l’âge et l’évolution du taux de mortalité annuel moyen avant et après avoir corrigé les décès portant des codes non spécifiques. Pour terminer, nous avons généré des projections en matière de mortalité routière à l’aide des codes corrigés et non corrigés, puis redistribué les données par intégration proportionnelle.

**Résultats:**

Nous avons analysé les données issues de 124 pays et territoires, avec au moins un an de données de mortalité entre 2015 et 2020. Deux pays et territoires mentionnaient plus de 20% de décès imputables à des causes inconnues ou mal définies ; trois pays signalaient des décès dus à des blessures non intentionnelles ; 21 faisaient état de blessures mortelles non intentionnelles et non spécifiées ; trois ne précisaient pas le type de véhicule impliqué dans les collisions mortelles ; et enfin, 30 pays rapportaient des accidents de la route mortels, indéterminés et involontaires. Après redistribution des décès munis de codes non spécifiques, le taux de mortalité routière a changé de plus de 50% dans 7% (5/73) à 18% (9/51) des pays et territoires.

**Conclusion:**

L’emploi de codes non spécifiques a entraîné une mauvaise estimation de la mortalité dans de nombreux pays. Nous recommandons aux responsables politiques et chercheurs actifs dans le domaine des traumatismes de tenir compte des éventuels pièges que recèle l’usage de données brutes ou non corrigées sur la mortalité routière, et leur conseillons de se fonder sur des données corrigées afin d’obtenir des estimations plus précises pour l’amélioration des programmes de sécurité routière.

## Resumen

**Objetivo:**

Evaluar la precisión y la fiabilidad de los datos sobre mortalidad por accidentes de tráfico registrados en la Base de datos sobre mortalidad de la Organización Mundial de la Salud. Asimismo, investigar cómo los datos incorrectos influyen en las estadísticas vitales sobre mortalidad utilizadas en programas de seguridad vial a nivel mundial.

**Métodos:**

Evaluamos la calidad de los datos en función del país y del territorio desde 2015 a 2020 calculando los porcentajes de cinco tipos de códigos de fallecimientos sin causa específica relacionados con la mortalidad por accidentes de tráfico. Comparamos la mortalidad por accidentes de tráfico en función de la edad, así como los cambios en el índice medio anual de mortalidad antes y después de corregir los fallecimientos con códigos no específicos. Generamos las proyecciones de mortalidad por accidentes de tráfico con códigos corregidos y sin corregir, y distribuimos de nuevo los datos utilizando el método proporcional.

**Resultados:**

Analizamos los datos correspondientes a 124 países y territorios que contaban con datos sobre mortalidad de al menos un año, del periodo comprendido entre 2015 y 2020. El número de países y territorios que notificaron más de un 20% de fallecimientos por causas imprecisas o desconocidas fue de 2; los países que notificaron fallecimientos por lesiones con intencionalidad indeterminada fueron 3; los países que notificaron fallecimientos por lesiones no intencionadas y no especificadas fueron 21; los países que notificaron fallecimientos no especificados en accidentes de transporte fueron 3; y los países que notificaron fallecimientos no especificados y no intencionados en accidentes de tráfico fueron 30. Tras redistribuir los fallecimientos con códigos no específicos, la mortalidad por accidentes de tráfico cambió en más de un 50% en un intervalo del 7% (5/73) al 18% (9/51) de los países y territorios.

**Conclusión:**

Los códigos no específicos dieron lugar a estimaciones imprecisas sobre mortalidad en numerosos países. Instamos a que los investigadores y los responsables de políticas de lesiones reconozcan las potenciales deficiencias que supone el hecho de basarse en datos no tratados ni corregidos, y a que, en lugar de eso, utilicen datos corregidos con el fin de garantizar estimaciones más precisas a la hora de mejorar los programas sobre seguridad vial.

## ملخص

**الغرض:**

تقييم دقة وموثوقية بيانات الوفيات الناجمة عن حوادث المرور على الطرق المسجلة في قاعدة بيانات الوفيات التابعة لمنظمة الصحة العالمية، والتحقيق في كيفية تأثير البيانات غير المصححة على إحصاءات الوفيات الحيوية المستخدمة في برامج السلامة المرورية في جميع أنحاء العالم.

**الطريقة:**

قمنا بتقييم جودة البيانات الخاصة بكل دولة وإقليم من عام 2015 إلى عام 2020، من خلال حساب نسب خمسة أنواع من رموز أسباب الوفاة غير المحددة المرتبطة بالوفيات الناجمة عن حوادث المرور. قمنا بمقارنة معدل الوفيات الناجمة عن حوادث المرور على الطرق، والمصححة حسب العمر، والتغيرات في متوسط معدل الوفيات السنوي، قبل وبعد تصحيح الوفيات برموز غير محددة. كما قمنا بتوليد توقعات الوفيات الناجمة عن حركة المرور على الطرق باستخدام الرموز المصححة وغير المصححة، وأعدنا توزيع البيانات باستخدام الطريقة المتناسبة.

**النتائج:**

قمنا بتحليل بيانات من 124 دولة وإقليمًا ذات بيانات الوفيات لمدة عام واحد على الأقل من 2015 إلى 2020. وكان عدد الدول والأقاليم التي أبلغت عن أكثر من %20 من الوفيات لأسباب غير محددة أو غير معروفة 2؛ وعدد الدول التي أبلغت عن الوفيات الناجمة عن إصابات غير محددة النية 3؛ وعدد الدول التي أبلغت عن وفيات غير محددة بسبب إصابات غير مقصودة 21؛ وعدد الدول التي أبلغت عن وفيات غير محددة في حوادث النقل 3؛ وعدد الدول التي أبلغت عن وفيات غير محددة وغير مقصودة بسبب حوادث المرور 30. وبعد إعادة توزيع الوفيات باستخدام رموز غير محددة، تغيرت الوفيات الناجمة عن حوادث المرور بأكثر من %50 في %7 (5/73)، إلى %18 (9/51) من البلدان والأقاليم.

**الاستنتاج:**

أدت الرموز غير المحددة إلى تقديرات غير دقيقة للوفيات في العديد من الدول. نحن نوصي كل من الباحثين وواضعي السياسات في مجال الإصابات بالإقرار بالمخاطر المحتملة للاعتماد على بيانات الوفيات الناجمة عن حوادث المرور المعتادة أو غير المصححة، واستخدام البيانات المصححة بدلاً من ذلك لضمان الحصول على تقديرات أكثر دقة عند تحسين برامج السلامة المرورية.

## 摘要

**目的:**

评估世界卫生组织死亡率数据库中包括的道路交通死亡率数据的准确性和可靠性，探讨未经校正的数据会如何影响世界各国交通安全计划中使用的重要死亡率统计数据。

**方法:**

通过计算与道路交通死亡率相关的五种非特异性死亡原因国家疾病分类代码的比例，本文评估了 2015 年至 2020 年期间特定国家和地区的数据质量，比较了在使用非特异性代码校正死亡人数前后按年龄调整的道路交通死亡率和年平均死亡率变化。本文利用经校正和未经校正的代码进行道路交通死亡率预测，并使用比例法重新分配记录为五种非特异性疾病分类代码的死亡数据。

**结果:**

作者分析了 124 个国家和地区在 2015 年至 2020 年期间至少一年的死亡率数据。报告不明或未知原因致死率达 20% 以上的国家和地区有 2 个；报告意图不明伤害致死达 20% 以上的国家有 3 个；报告非特定意外伤害死亡达 20% 以上的国家有 21 个；报告非特定交通事故死亡达 20% 以上的国家有 3 个；以及报告非特定意外道路交通死亡达 20% 以上的国家有 30 个。使用非特异性代码重新分配死亡人数后，有 7% (5/73) 至 18% (9/51) 的国家和地区的道路交通死亡率变化幅度高达 50% 以上。

**结论:**

在许多国家，非特异性代码导致死亡率估不准确。我们建议，伤害相关研究人员和政策制定者应认识到依赖原始或未经校正的道路交通死亡率数据可能存在潜在风险，并在改进交通安全规划时选用经校正的数据，以确保获得更准确的估值。

## Резюме

**Цель:**

Оценить точность и достоверность данных о смертности в результате ДТП, зарегистрированных в Базе данных по смертности Всемирной организации здравоохранения, и выяснить, как нескорректированные данные влияют на статистику смертности, используемую в программах безопасности дорожного движения во всем мире.

**Методы:**

Проведена оценка качества данных по странам и территориям в период с 2015 по 2020 год путем расчета доли пяти типов неспецифических кодов причин смертности в результате ДТП. Сравнивались данные о смертности в результате ДТП с поправкой на возраст и изменения среднегодового коэффициента смертности до и после коррекции случаев смерти с неспецифическими кодами. Составлены прогнозы смертности в результате ДТП как с исправленными, так и с неисправленными кодами, и произведено перераспределение данных с использованием метода пропорциональности.

**Результаты:**

Проведен анализ данных из 124 стран и территорий, по которым имеются данные о смертности за 2015–2020 гг. как минимум за один год. Количество стран и территорий, сообщивших о более чем 20% смертей по неустановленной или неизвестной причине, составило 2; стран, сообщивших о смертях от травм с неустановленным намерением, – 3; стран, сообщивших о неуточненных неумышленных смертях от травм, – 21; стран, сообщивших о неуточненных неумышленных смертях в транспортных авариях, – 3; стран, сообщивших о неуточненных неумышленных смертях в результате ДТП, – 30. После перераспределения случаев смертности с использованием неспецифических кодов уровень смертности в результате ДТП изменился более чем на 50% в 7% (5/73) до 18% (9/51) стран и территорий.

**Вывод:**

Использование неспецифических кодов привело к неточным оценкам уровня смертности во многих странах. Исследователям травматизма и представителям власти рекомендуется признать потенциальные недостатки использования необработанных или нескорректированных данных о смертности в результате ДТП и вместо них использовать скорректированные данные для получения более точных оценок при совершенствовании программ по безопасности движения.

## Introduction

Globally, road traffic crashes cause approximately 1.3 million preventable deaths and 50 million injuries each year.^1^ In 2021, the United Nations (UN) General Assembly resolution 74/299 committed to reduce by 50% the number of deaths and injuries caused by road traffic crashes worldwide by 2030.^2^

Accurate and reliable mortality data serve as the foundation for tracking advancements and forecasting future progress in attaining the objectives of global road traffic safety initiatives. Among available data sets for this purpose, arguably the most comprehensive is the World Health Organization (WHO) Mortality Database, which compiles yearly mortality data reported by Member States from their civil registration and vital statistics systems.^3^ The WHO Mortality Database is a data repository which is used for comparative epidemiological studies of all-cause mortality rates^3^ and estimations of disease burden and temporal trend by international organizations like WHO, the World Bank, the Global Health Estimates study group, the Global Burden of Disease (GBD) study group, and many other health researchers.^4^^–^^7^ One limitation of the WHO Mortality Database is that it includes a substantial number of nonspecific death codes that do not comply with the principles of the International Statistical Classification of Diseases and Related Health Problems, 10^th^ revision (ICD-10).^8^ Despite this notable limitation, raw and uncorrected data is still being used by researchers for analysis.^9^^,^^10^

Efforts have been made by the Global Health Estimates study group to correct data quality challenges arising from incorrect death codes.^5^ Their research showed that data users can provide comprehensive and comparable cause of death estimates by assessing and redistributing nonspecific ICD-10 codes.^5^^,^^11^ Unfortunately, the Global Health Estimates study group did not publish a detailed quality analysis of raw road traffic mortality data, nor did they quantify its influence.

The GBD study group also assessed and redistributed nonspecific ICD-10 codes for 204 countries and territories between 1990–2019. However, this team did not publicly release information on the coding quality of the mortality data they used, nor did they publish a comprehensive assessment of the quality of that data.^6^

A few researchers have used related data sets from specific Member States, namely Brazil and South Africa, to explore the impact of death coding quality.^12^^–^^15^ However, we did not find any research which systematically analysed the quality of road traffic mortality data from the WHO Mortality Database, or quantified its influence on monitoring progress towards global health goals or projecting future global road traffic safety trends. 

The main goal of this study is to assess the availability and coding quality of road traffic mortality data from the WHO Mortality Database, using data from the years 2015 to 2020. Additionally, we seek to investigate the influence of coding quality on data used to monitor the progress of global road traffic safety initiatives.

## Methods

### Data source

We retrieved all mortality data from the WHO Mortality Database (as of 27 February 2023).^3^ We used complete country data sets from 2015 to 2020 for our analysis. All identified deaths were coded using ICD-10. We used country-level population data from the UN World Population Prospects 2022 estimates and projections.^16^

### Indicators for data quality

We used two indicators from previously published literature to approximate data quality for global road traffic mortality data: (i) data availability; and (ii) coding quality.^5^^,^^8^^,^^17^^–^^20^

To assess data availability, we used the presence or absence of road traffic mortality data in the WHO Mortality Database to reflect on overall data availability. We quantified data availability in number of years since 2015 by determining whether mortality data were available for 0 years (unavailable), 1 year, 2 years, 3 years, 4 years, 5 years or 6 years.

**Fig. 1:**
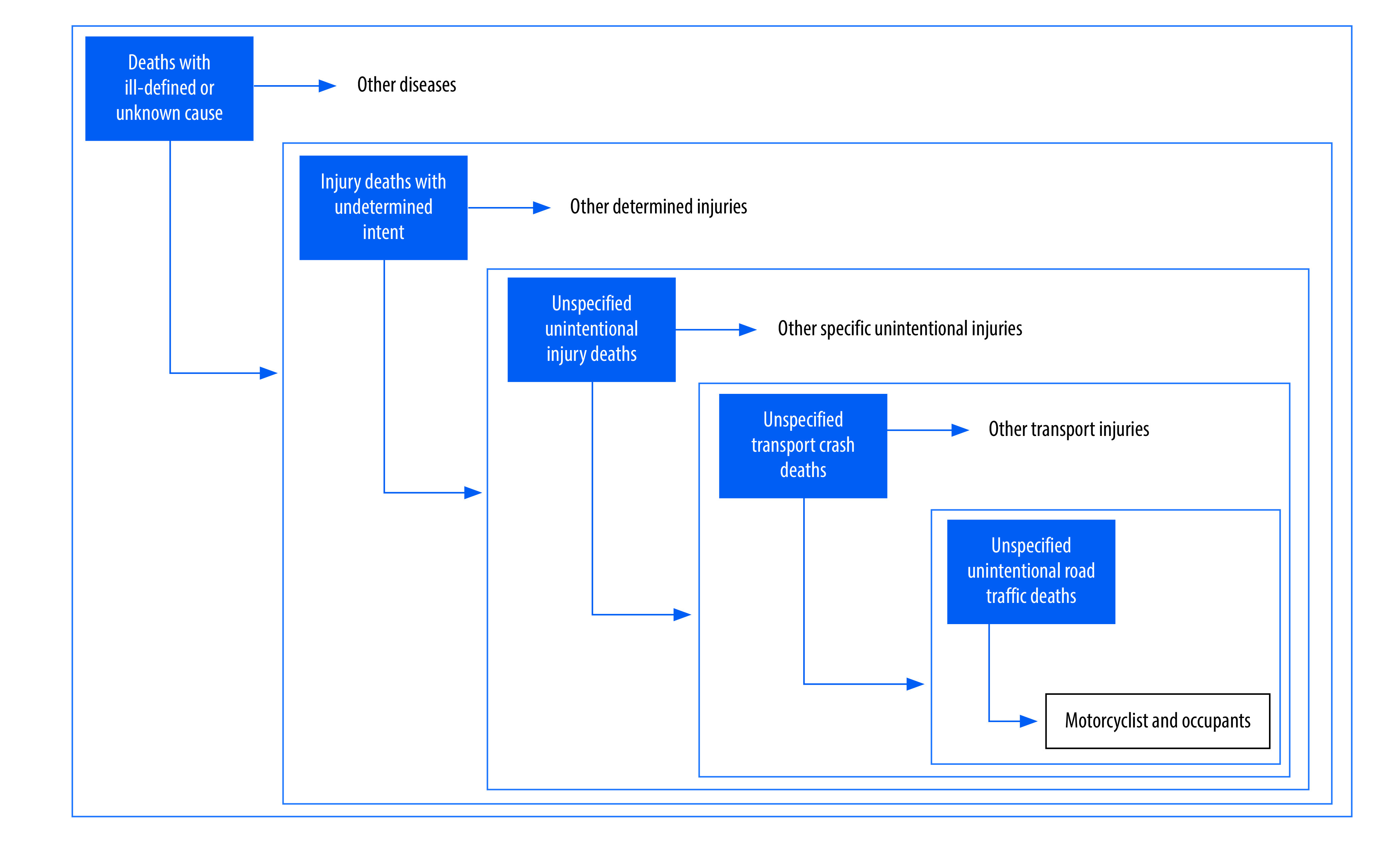
Categorization and distribution of deaths with nonspecific ICD-10 codes for assessing coding quality of road traffic mortality data

To assess coding quality, we calculated the percentages of nonspecific codes in five predefined categories: (i) total deaths; (ii) injury deaths; (iii) unintentional injury deaths; (iv) transport crash deaths; and (v) unintentional road traffic deaths. To identify nonspecific ICD-10 codes that we could use to quantify the coding quality of road traffic mortality data, we searched previous literature,^8^^,^^18^^–^^20^ and mapped the following codes to the above categories: (i) deaths with ill-defined or unknown cause (ICD-10 codes: R95, R96, R98 and R99); (ii) injury deaths with undetermined intent (ICD-10 codes: Y34, Y87.2 and Y89.9); (iii) unspecified unintentional injury deaths (ICD-10 codes: X59); (iv) unspecified transport crash deaths (ICD-10 codes: V99 and Y85.9); and (v) unspecified unintentional road traffic deaths (ICD-10 codes: V87.0–V87.8 and V89.2; Fig. 1). 

ICD: International statistical classification of diseases and related health problems, 10th revision.

We followed the ICD classification framework^8^ step by step and classified the percentages of nonspecific codes into five groups: (i) 0%–20%; (ii) 21%–40%; (iii) 41%–60%; (iv) 61%–80%; and (v) 81%–100%.

### Redistribution of deaths

We used the proportionate method^21^ to redistribute deaths with nonspecific codes to cause-specific codes. This method assumes that deaths with nonspecific codes follow the same all-cause distribution as deaths with cause-specific codes, and can therefore be redistributed to all cause-specific deaths. Furthermore, the proportionate method is preferable when there is limited covariate data available for use.^22^ Previous studies found that corrected results for road injuries, obtained through the proportionate method, were more accurate in approximating true values compared to the use of more intricate methods.^19^

### Statistical analysis

According to the ICD-10 manual,^8^ the first four types of nonspecific ICD-10 codes tend to underestimate overall road traffic mortality and user-specific road traffic mortality, while the last type, unspecified unintentional road traffic deaths, tends to specifically underestimate road traffic mortality for occupants and motorcyclists. We therefore redistributed deaths with the first four types of nonspecific ICD-10 codes to calculate the age-adjusted overall road traffic mortality and user-specific road traffic mortality for pedal cyclists and pedestrians, and used deaths with all five types of nonspecific codes to calculate the age-adjusted road traffic mortality for occupants and motorcyclists (Fig. 1). When the proportion of deaths with nonspecific codes was < 30%, 30%–49%, 50%–69% or ≥ 70% in a particular year, we used the proportion of cause-specific deaths in the same year (the study year); a three-year period (the preceding year, the study year, and the next year); a five-year period (two preceding years, the study year, and the next two years); or a seven-year period (three preceding years, the study year, and the next three years) to redistribute deaths with nonspecific codes. When relevant data for the preceding and/or following years were unavailable, we used all available data to compute corrections.

To assess the influence of deaths with nonspecific codes on overall road safety data, we compared age-adjusted overall road traffic mortality rates and user-specific road traffic mortality rates for occupants, motorcyclists, pedal cyclists and pedestrians, both before and after correcting (redistributing) deaths with nonspecific ICD-10 codes. Age-adjusted mortality rates were calculated using the new WHO world standard population values.^23^ The ratio of corrected/uncorrected mortality rates was then used to quantify the proportionate influence of deaths with nonspecific codes on road safety statistics.

To assess the impact of nonspecific ICD-10 codes on trends in road traffic crash data, we compared the average annual change rate for road traffic deaths before and after correcting and redistributing deaths with nonspecific codes. Based on the robustness to extreme values reported in the previous analysis,^24^ we selected the geometric mean method to calculate the average annual change rate of road traffic mortalities. In addition, we used the average annual change rate of road traffic deaths to project the number of road traffic deaths in both 2021 and 2030; and compared theprojected percent change in road traffic deaths between the years 2021 to 2030.

All data were analysed using SAS version 9.4 (SAS, Cary, United States of America (USA)), R version 4.3.1 (R Foundation, Vienna, Austria) and Microsoft Office 2016 (Microsoft, Redmond, USA). Our study protocol was approved by the Medical Ethics Committee of Central South University, Changsha, China, on 25 January 2021 (No. XYGW-2021–06). 

## Results

### Data availability

**Fig. 2:**
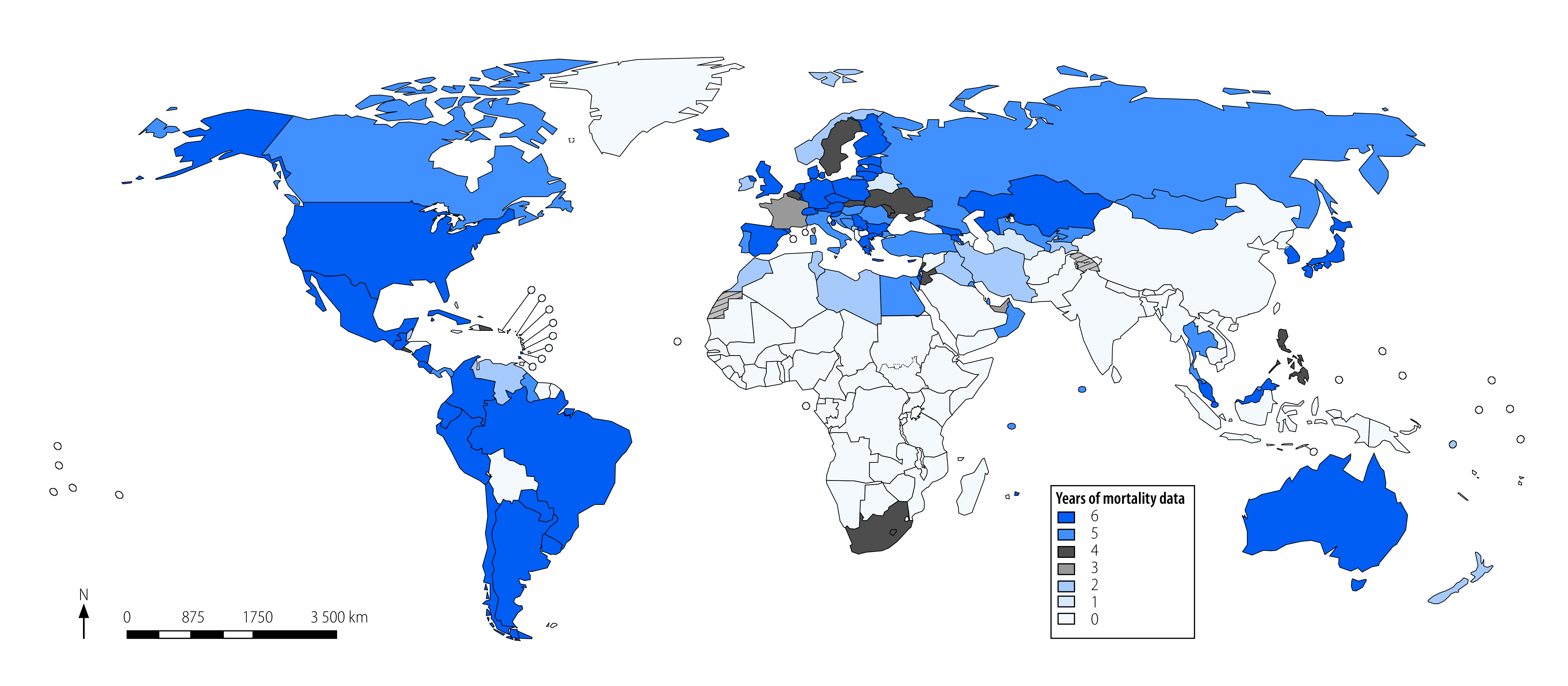
Overall availability of road traffic mortality data in the WHO Mortality Database, 2015–2020

As of 27 February 2023, 124 countries and territories reporting to the WHO Mortality Database had at least one complete year of mortality data available between 2015 and 2020 (Fig. 2). The WHO European Region had the most years of available data (248 years), while the South-East Asia Region had the fewest (10 years; Table 1).

WHO: World Health Organization.

**Table 1:** Years of available road traffic mortality data from six WHO regions in the WHO Mortality Database, 2015–2020

WHO Region	No of countries or territories	Years of available data
African Region	47	15
Region of the Americas	35	144
Eastern Mediterranean Region	21	41
European Region	53	248
South-East Asia Region	11	10
Western Pacific Region	27	48

WHO: World Health Organization.

### Coding quality

**Fig. 3:**
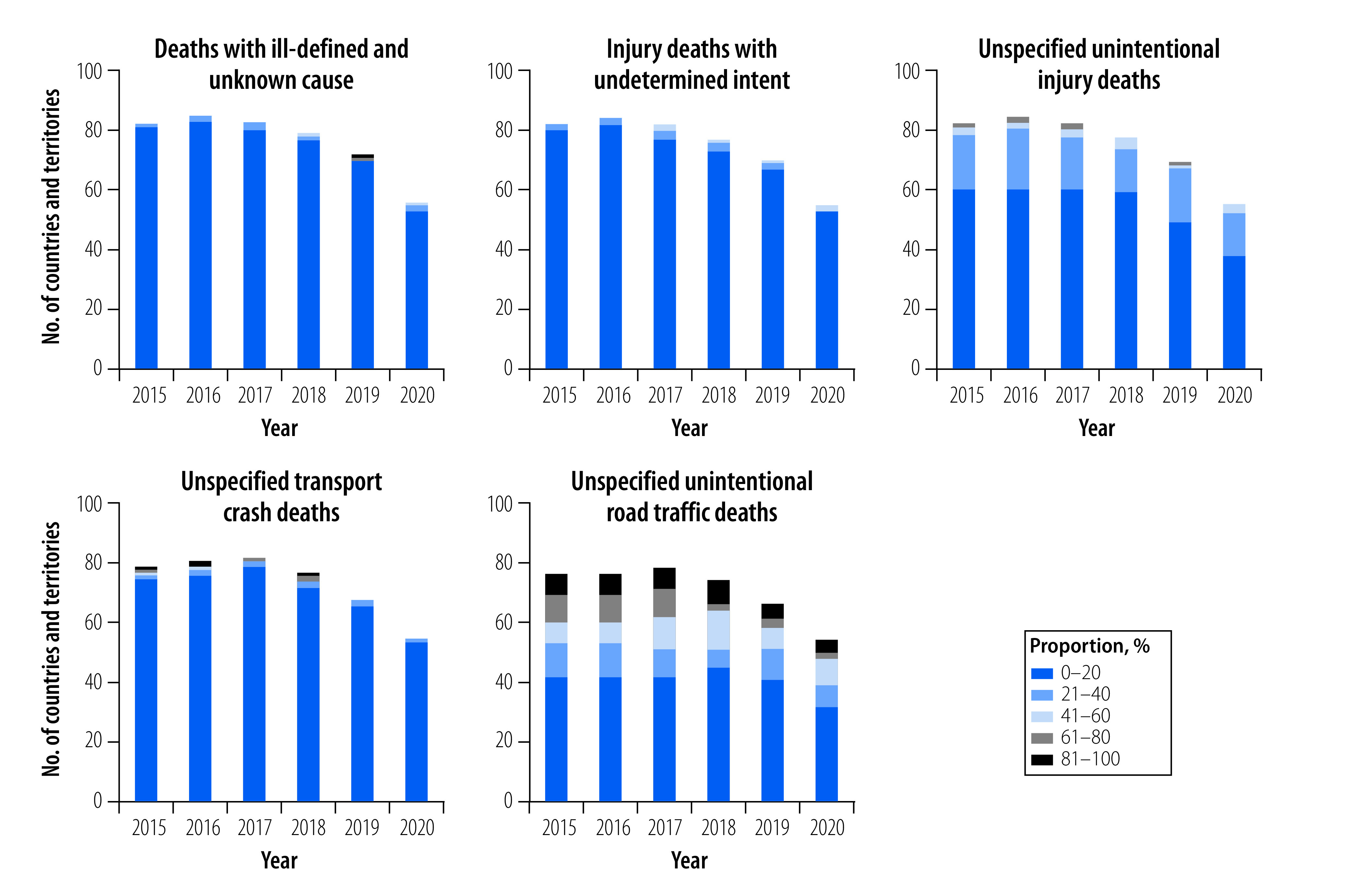
Proportion of mortalities for five types of nonspecific ICD-10 codes, 97 countries and territories, 2015–2020

Out of the 97 countries and territories using the 4-digit ICD-10 coding system for reporting cause of death, the average number of countries and territories per year reporting over 20% nonspecific mortalities for each type of nonspecific code were: (i) two countries and territories had codes of ill-defined and unknown cause; (ii) three countries and territories had codes of injury deaths with undetermined intent; (iii) 21 countries and territories had codes of unspecified unintentional injury deaths; (iv) three countries and territories had codes of unspecified transport crash deaths; and (v) 30 countries and territories had codes of unspecified unintentional road traffic deaths (Fig. 3).

ICD: International statistical classification of diseases and related health problems, 10th revision.

Notes: We obtained data from the WHO Mortality Database. The ICD-10 codes for each type are: R95, R96, R98 and R99 for deaths with ill-defined or unknown cause; Y34, Y87.2 and Y89.9 for injury deaths with undetermined intent; X59 for unspecified unintentional injury deaths; V99 and Y85.9 for unspecified transport crash deaths; and V87.0–V87.8 and V89.2 for unspecified unintentional road traffic deaths. The number of countries or territories reporting data changed each year and was consistently fewer than the total count of 97 countries or territories that reported data at some point between 2015 and 2020.

### Impact of coding quality

**Fig. 4:**
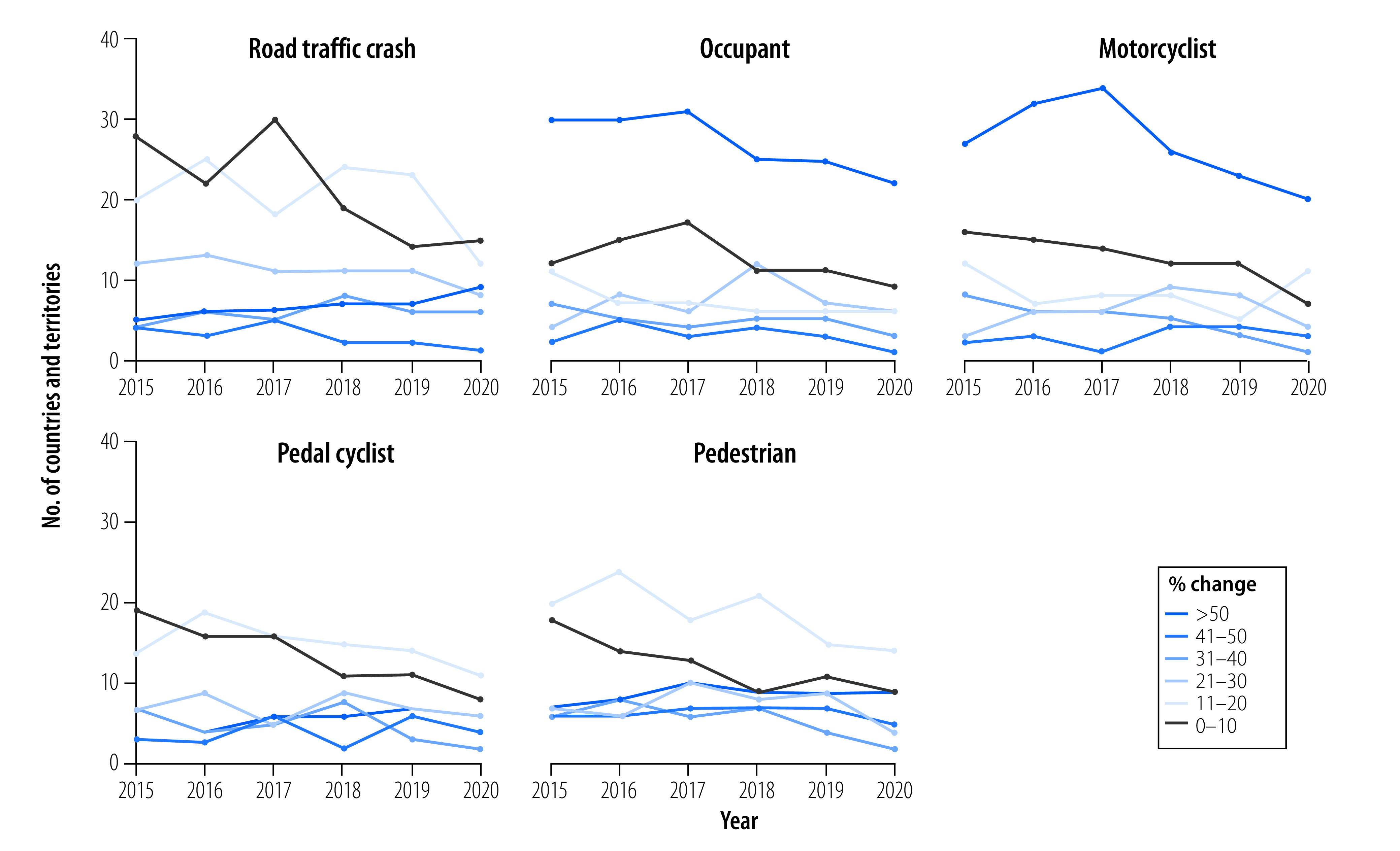
Percentage change in overall and user-specific age-adjusted road traffic mortality rates after correcting nonspecific ICD-10 codes, 93 countries and territories, 2015–2020

After correcting for mortality data with nonspecific ICD-10 codes, shifts were observed in age-adjusted overall and user-specific road traffic mortality for most of the 93 countries and territories with available data. Notably, over the 6-year period from 2015 to 2020, on average seven countries and territories experienced a greater than 50% increase in age-adjusted overall road traffic mortality (Fig. 4). 

ICD: International statistical classification of diseases and related health problems, 10th revision.

Note: The number of countries or territories reporting data changed each year and was consistently fewer than the total count of 93 countries or territories that reported data at some point between 2015 and 2020.

For occupants, an average of 27 countries and territories per year experienced a greater than 50% increase in age-adjusted mortality. For motorcyclists, an average of 27 countries and territories experienced a greater than 50% increase in age-adjusted mortality. For pedal cyclists, only six countries and territories on average saw a greater than 50% increase in age-adjusted mortality. Lastly, for pedestrians, nine countries and territories on average experienced a greater than 50% increase in age-adjusted mortality.

We calculated the average annual change rate for the 76 countries and territories presenting complete mortality data for two or more years between 2015 and 2020. Before correction, the average annual change rate of road traffic deaths was greater than zero (positive) for 16 countries and territories; at zero (neutral) for two territories; and less than zero (negative) for 58 countries and territories (Table 2). After correcting deaths with nonspecific ICD-10 codes, the average annual change rate increased from zero to greater than zero (positive) for Bermuda; decreased from zero to less than zero (negative) for the United States Virgin Islands; and reversed from less than zero (negative) to greater than zero (positive) in El Salvador, Kuwait, Mauritius, Poland and Tunisia. Notably, rates of change shifted by more than 10% for Lebanon (54.5% to 34.8%); Peru (−20.7% to −37.9%); Tunisia (−4.8% to 7.8%); and Oman (−27.9% to −16.1%).

**Table 2:** Average annual change of road traffic mortalities before and after correcting nonspecific ICD-10 codes, 76 countries and territories, 2015–2020

Country or territory, by WHO region	Average annual change, %	
	Uncorrected	Corrected
**African Region**		
Mauritius	−1.3	0.4
**Region of the Americas**		
Argentina	−13.3	−10.5
Aruba	−14.6	−14.5
Bermuda	0.0	0.1
Brazil	−3.6	−3.1
Canada	−6.9	−6.5
Chile	−5.6	−5.7
Colombia	−4.3	−3.5
Costa Rica	−12.1	−10.5
Cuba	−8.7	−8.3
Dominica	−100.0	−100.0
Dominican Republic	7.4	7.3
Ecuador	−2.0	−0.4
El Salvador	−2.5	4.2
Grenada	−38.1	−36.5
Guatemala	10.8	11.2
Guyana	−1.7	−1.2
Mexico	−2.1	−1.1
Montserrat	−100.0	−100.0
Nicaragua	5.5	5.0
Panama	−6.6	−5.8
Paraguay	−0.2	−0.8
Peru	−20.7	−37.9
Puerto Rico	−1.9	−5.2
Saint Vincent and Grenadines	−27.5	−26.4
United States	2.2	1.9
Uruguay	−2.0	−1.2
Virgin Islands	0.0	−7.4
**Eastern Mediterranean Region**		
Kuwait	−1.3	0.4
Lebanon	54.5	34.8
Oman	−27.9	−16.1
Tunisia	−4.8	7.8
**European Region**		
Armenia	0.5	3.8
Austria	0.2	4.2
Belgium	−9.5	−8.9
Bulgaria	−8.0	−9.3
Croatia	−8.9	−8.2
Cyprus	−6.2	−4.1
Czechia	−6.5	−5.8
Denmark	−3.9	−5.5
Estonia	3.4	3.1
France	−2.3	−1.6
Georgia	−6.1	−5.9
Germany	−7.0	−4.5
Greece	−4.9	−3.9
Hungary	−0.7	−0.8
Iceland	6.1	7.8
Ireland	−14.0	−12.0
Israel	−7.9	−8.2
Italy	−2.1	−0.5
Kazakhstan	−8.9	−8.7
Kyrgyzstan	−4.1	−4.7
Latvia	−6.3	−6.1
Lithuania	−5.6	−5.6
Luxembourg	−5.6	−6.8
Malta	24.3	23.5
Netherlands (Kingdom of the)	−0.5	−1.1
Poland	−3.1	1.0
Portugal	1.9	2.6
Republic of Moldova	−7.9	−6.6
Romania	−1.0	−1.5
Slovakia	6.0	5.7
Spain	−6.0	−6.6
Sweden	8.2	8.3
Switzerland	−6.8	−5.6
Türkiye	−7.4	−5.9
United Kingdom	−4.8	−5.0
**South-East Asia Region**		
Maldives	−6.9	−5.5
Thailand	12.8	4.5
**Western Pacific Region**		
Australia	−1.8	−1.9
Brunei Darussalam	−15.3	−16.0
China, Hong Kong SAR	16.5	17.4
Japan	−8.7	−7.8
Mongolia	1.9	2.0
Republic of Korea	−7.4	−7.7
Singapore	−10.2	−10.0

ICD-10: International statistical classification of diseases and related health problems, 10th revision; SAR: Special administrative region; WHO: World Health Organization.

Note: We included data from 76 countries or territories that had mortality data for two or more years between 2015 and 2020.

Before correcting mortality data with nonspecific ICD-10 codes, the number of road traffic deaths was projected to increase in 16 (21%) countries and territories, remain unchanged in two (3%) territories, and decrease in 58 (76%) countries and territories. In contrast, the number of road traffic deaths was projected to increase in 22 (29%) countries and territories, and decrease in 54 (71%) countries and territories after correction. 

Prior to correction, 22 countries or territories also appeared to be on track to achieve global safety targets set for the *Global plan for the decade of action for road safety 2021–2030*.^2^ However, after correction, Republic of Moldova and Türkiye shifted from the ‘achieve the target’ category to the ‘unable to achieve the target’ category. Additionally, the projected percentage change in road traffic mortality rates between 2021 and 2030 underwent significant alterations of more than 50% for four countries and territories: Lebanon (changing from 4904.7% to 1373.9%); Thailand (changing from 194.8% to 48.7%); Tunisia (changing from −35.5% to 97.1%); and El Salvador (changing from −20.4% to 44.8%; Table 3).

**Table 3:** Projected percent changes in road traffic mortalities before and after correcting nonspecific ICD-10 codes, 76 countries and territories, 2021–2030

Country or territory, by WHO region	Projected changes in road traffic mortality, %	
	Uncorrected	Corrected
**African Region**		
Mauritius	−11.2	3.3
**Region of the Americas**		
Argentina	−72.3	−63.3
Aruba	−75.8	−75.7
Bermuda	0.0	0.8
Brazil	−28.0	−24.5
Canada	−47.5	−45.2
Chile	−40.6	−40.8
Colombia	−32.9	−27.3
Costa Rica	−68.8	−63.3
Cuba	−55.9	−54.1
Dominica	−100.0	−100.0
Dominican Republic	90.5	88.0
Ecuador	−16.9	−3.6
El Salvador	−20.4	44.8
Grenada	−98.7	−98.3
Guatemala	150.8	159.0
Guyana	−14.6	−10.0
Mexico	−17.4	−9.8
Montserrat	−100.0	−100.0
Nicaragua	61.9	55.6
Panama	−46.1	−41.7
Paraguay	−2.1	−7.2
Peru	−87.6	−98.6
Puerto Rico	−16.0	−38.2
Saint Vincent and Grenadines	−94.5	−93.6
United States	21.2	18.3
Uruguay	−16.5	−10.6
Virgin Islands	0.0	−50.0
**Eastern Mediterranean Region**		
Kuwait	−10.9	3.9
Lebanon	4904.7	1373.9
Oman	−94.7	−79.3
Tunisia	−35.5	97.1
**European Region**		
Armenia	4.6	40.3
Austria	1.6	44.2
Belgium	−59.3	−56.8
Bulgaria	−52.9	−58.4
Croatia	−56.8	−53.8
Cyprus	−44.0	−31.1
Czechia	−45.3	−41.5
Denmark	−30.2	−39.8
Estonia	35.4	32.2
France	−18.6	−13.6
Georgia	−43.2	−42.1
Germany	−47.9	−34.0
Greece	−36.4	−30.0
Hungary	−5.8	−6.9
Iceland	69.9	96.3
Ireland	−74.2	−68.2
Israel	−52.3	−53.6
Italy	−17.5	−4.3
Kazakhstan	−56.7	−56.0
Kyrgyzstan	−31.5	−35.0
Latvia	−44.2	−43.2
Lithuania	−40.3	−40.5
Luxembourg	−40.4	−46.8
Malta	609.2	567.1
Netherlands (Kingdom of the)	−4.5	−9.2
Poland	−24.9	9.2
Portugal	18.1	26.2
Republic of Moldova	−52.3	−46.1
Romania	−9.0	−12.3
Slovakia	68.4	64.1
Spain	−42.9	−45.7
Sweden	103.1	104.8
Switzerland	−47.0	−40.7
Türkiye	−50.0	−42.3
United Kingdom	−36.1	−36.7
**South-East Asia Region**		
Maldives	−47.7	−39.7
Thailand	194.8	48.7
**Western Pacific Region**		
Australia	−15.1	−16.2
Brunei Darussalam	−77.4	−79.1
China, Hong Kong SAR	296.2	322.4
Japan	−55.7	−51.6
Mongolia	18.7	19.3
Republic of Korea	−50.2	−51.4
Singapore	−61.8	−61.2

ICD-10: International statistical classification of diseases and related health problems, 10th revision; SAR: Special administrative region; WHO: World Health Organization.

Note: We included data from 76 countries or territories that had mortality data for two or more years between 2015 and 2020.

## Discussion

Our analysis generated three main sets of findings. First, the number of countries and territories reporting mortality data to WHO for the 2015–2020 period was low, with some WHO regions underrepresented. Previous studies reported that mortality data were available for at least one year in the WHO Mortality Database for 115 countries and territories from 1950 to 2003,^25^ and for 83 countries and territories from 2000 to 2009.^18^ Our findings suggest that the availability of mortality data in the WHO Mortality Database has not improved over the period from 2015 to 2020, as we only observed 52 countries and territories reporting complete data for all 6 years. Possible reasons for poor data availability include: (i) some countries and territories have not established reliable vital statistics systems to collect mortality data;^26^ (ii) vital registration systems in some countries and territories are disrupted by war or political unrest;^27^^,^^28^ (iii) several countries and territories do not use standard ICD codes to record mortality data;^29^ (iv) some countries and territories lack adequate resources and qualified coders to reliably complete death certificates and gather mortality data;^30^^,^^31^ and (v) some countries and territories refuse to submit their data to WHO, perhaps due to concerns about data misinterpretation or misuse, intentional or unintentional privacy disclosure, or loss of data ownership.^32^^,^^33^

Second, about three fifths of countries and territories reported more than 20% of nonspecific ICD-10 codes for road traffic deaths, particularly for deaths coded with unspecified unintentional injury deaths and unspecified unintentional road traffic deaths. This unsatisfactory coding quality of data corroborates the results of a previous study,^18^ which highlighted the link between less cause specificity and the proportion of unspecified unintentional road injury deaths across six types of ICD codes in WHO mortality data, as updated on 21 April 2009.

Third, we found that nonspecific ICD-10 codes underestimated road traffic mortality rates by more than 20% for 70 countries and territories, influencing our ability to accurately monitor progress and project future outcomes for global road safety development targets. These findings are generally consistent with previous reports for individual countries like Mexico,^34^ Republic of Korea^35^ and the USA,^36^ that suggest nonspecific ICD-10 coding has negatively influenced fatal injury statistics and trends. After correcting mortality data with nonspecific ICD-10 codes, we found that indicators such as road traffic mortality rates, progress monitoring figures, and projections of future trends in global road safety targets were strongly affected. In addition, we found that the average annual change rates shifted from negative to positive values upon correction for five countries. Additionally, the projected future rate changes changed from ‘achieve the target’ to ‘unable to achieve the target’ status for two countries. 

Irrespective of the negative outcomes noted above, coding quality has improved in many countries and territories in recent years; despite a few exceptions (for example, Brazil and the United Republic of Tanzania) where coding quality has declined due to insufficient numbers of certified medical coders and/or increased workloads per coder.^37^^,^^38^

Our findings have several policy implications. First, to enhance the WHO Mortality Database's data availability and its significance in decision-making, policy-making and scientific research, Member States possessing mortality data need to prioritize data-sharing for national and global health. These Member States should promptly report the data to WHO as required. For those without reliable mortality data, implementing the *WHO civil registration and vital statistics strategic implementation plan 2021–2025* will help improve civil registration and vital statistics capacity, and the SCORE for Health Data Technical Package could be used as a technical tool in these countries.^39^^,^^40^

Second, we propose a series of measures to enhance coding quality in countries and territories facing poor quality death coding. These actions include standard training of death certificate coders based on standards from the Data for Health Initiative.^41^ Additionally, using procedures outlined in the Analysis of National Causes of Death for Action tool can improve the accuracy of data coding.^42^ Furthermore, the development of artificial intelligence-driven automatic coding tools with high predictive performance may help improve the quality of coding by overcoming shortages of qualified death certificate coders.^43^ For example, deep semantic matching or classification models based on analogical reasoning^44^ and federated learning^45^ could automatically code deaths, with human coders only used to confirm artificial intelligence-selected cases. Resources should be provided by international donors to countries and territories which cannot afford to implement these changes, or that understandably prioritize other national efforts.

Lastly, injury researchers and policy-makers should exercise caution while using raw mortality data, as recommended by WHO on its database website.^3^ More emphasis should be placed on rigorously evaluating data quality, making critical corrections to raw data, and interpreting the results with careful consideration.

This study has several limitations. First, we focused only on nonspecific mortality coding and were unable to validate other quality problems such as underreporting, overreporting or misclassification across all death causes. Thus, our findings do not necessarily reflect the importance of all data quality problems on statistical interpretation.

Second, we were unable to investigate factors influencing coding quality in each country and territory due to the absence of relevant policy documents outlining data collection strategies. Setting up a large-scale research programme would be beneficial to explore these factors and develop viable solutions to inconsistencies in the data. Last, the proportionate method we used to redistribute mortalities with nonspecific codes relies on the assumption that deaths with nonspecific ICD-10 codes follow the same cause distribution pattern as deaths with specific codes.^21^ If this assumption is violated, our corrected results may be invalid.

In conclusion, as injury researchers and policy-makers, it is imperative that we acknowledge the potential pitfalls of relying on raw or uncorrected road traffic mortality data and approach analytical findings with utmost caution. Only through rigorous assessment and interpretation can we understand the complexities of road traffic safety data and make informed decisions to improve global road safety.

## References

[1] Road traffic injuries. Geneva: World Health Organization; 2022. Available from: https://www.who.int/news-room/fact-sheets/detail/road-traffic-injuries [cited 2023 May 20].

[2] Global plan for the decade of action for road safety 2021–2030. Geneva: World Health Organization; 2021. Available from: https://www.who.int/publications/m/item/global-plan-for-the-decade-of-action-for-road-safety-2021-2030 [cited 2023 May 20].

[3] WHO mortality database. Geneva: World Health Organization; 2022. Available from: https://www.who.int/data/data-collection-tools/who-mortality-database [cited 2023 May 20].

[4] Trends in maternal mortality 2000 to 2017. New York: United Nations Population Fund; 2019. Available from: https://www.unfpa.org/featured-publication/trends-maternal-mortality-2000-2017 [cited 2023 May 20].

[5] WHO methods and data sources for country-level causes of death 2000–2019. Geneva: World Health Organization; 2020. Available from: https://www.who.int/docs/default-source/gho-documents/global-health-estimates/ghe2019_cod_methods.pdf?sfvrsn=37bcfacc_5 [cited 2023 May 20].

[6] Vos T, Lim SS, Abbafati C, Abbas KM, Abbasi M, Abbasifard M, et al. GBD 2019 Diseases and Injuries Collaborators. Global burden of 369 diseases and injuries in 204 countries and territories, 1990-2019: a systematic analysis for the Global Burden of Disease Study 2019. Lancet. 2020 Oct 17;396(10258):1204–22. 10.1016/S0140-6736(20)30925-9 33069326 PMC7567026

[7] Lampropoulos IC, Malli F, Sinani O, Gourgoulianis KI, Xiromerisiou G. Worldwide trends in mortality related to Parkinson’s disease in the period of 1994-2019: Analysis of vital registration data from the WHO Mortality Database. Front Neurol. 2022 Oct 4;13:956440. 10.3389/fneur.2022.956440 36267881 PMC9576872

[8] International statistical classification of diseases and related health problems, 10th revision. Geneva: World Health Organization; 2019. Available from: https://icd.who.int/browse10/2019/en [cited 2023 May 20].

[9] Huang J, Deng Y, Tin MS, Lok V, Ngai CH, Zhang L, et al. Distribution, risk factors, and temporal trends for lung cancer incidence and mortality: a global analysis. Chest. 2022 Apr;161(4):1101–11. 10.1016/j.chest.2021.12.655 35026300

[10] Baum P, Winter H, Eichhorn ME, Roesch RM, Taber S, Christopoulos P, et al. Trends in age- and sex-specific lung cancer mortality in Europe and Northern America: Analysis of vital registration data from the WHO Mortality Database between 2000 and 2017. Eur J Cancer. 2022 Aug;171:269–79. 10.1016/j.ejca.2022.05.011 35738973

[11] WHO methods and data sources for global burden of disease estimates 2000-2019. Geneva: World Health Organization; 2020. Available from: https://cdn.who.int/media/docs/default-source/gho-documents/global-health-estimates/ghe2019_daly-methods.pdf?sfvrsn=31b25009_7 [cited 2023 May 20].

[12] Huang H, Yin Q, Schwebel DC, Ning P, Hu G. Availability and consistency of health and non-health data for road traffic fatality: Analysis of data from 195 countries, 1985-2013. Accid Anal Prev. 2017 Nov;108:220–6. 10.1016/j.aap.2017.08.033 28915503

[13] Nittayasoot N, Peterson AB, Thammawijaya P, Parker EM, Sathawornwiwat A, Boonthanapat N, et al. Evaluation of a hospital-based injury surveillance system for monitoring road traffic deaths in Phuket, Thailand. Traffic Inj Prev. 2019;20(4):365–71. 10.1080/15389588.2019.1581924 31050566 PMC6584949

[14] Chokotho LC, Matzopoulos R, Myers JE. Assessing quality of existing data sources on road traffic injuries (RTIs) and their utility in informing injury prevention in the Western Cape Province, South Africa. Traffic Inj Prev. 2013;14(3):267–73. 10.1080/15389588.2012.706760 23441945

[15] Mandacaru PMP, Andrade AL, Rocha MS, Aguiar FP, Nogueira MSM, Girodo AM, et al. Qualifying information on deaths and serious injuries caused by road traffic in five Brazilian capitals using record linkage. Accid Anal Prev. 2017 Sep;106:392–8. 10.1016/j.aap.2017.06.018 28728061

[16] World population prospects 2022. New York: United Nations; 2022. Available from: https://population.un.org/wpp/Download/Archive/Standard/ [cited 2023 May 20].

[17] Mikkelsen L, Phillips DE, AbouZahr C, Setel PW, de Savigny D, Lozano R, et al. A global assessment of civil registration and vital statistics systems: monitoring data quality and progress. Lancet. 2015 Oct 3;386(10001):1395–406. 10.1016/S0140-6736(15)60171-4 25971218

[18] Bhalla K, Harrison JE, Shahraz S, Fingerhut LA; Global Burden of Disease Injury Expert Group. Availability and quality of cause-of-death data for estimating the global burden of injuries. Bull World Health Organ. 2010 Nov 1;88(11):831–838C. 10.2471/BLT.09.068809 21076564 PMC2971504

[19] Bhalla K, Harrison JE. GBD-2010 overestimates deaths from road injuries in OECD countries: new methods perform poorly. Int J Epidemiol. 2015 Oct;44(5):1648–56. 10.1093/ije/dyv019 25817298

[20] External cause of injury mortality matrix for ICD-10. Atlanta: Centers for Disease Control and Prevention, US Department of Health & Human Services; 2002. Available from: https://www.cdc.gov/nchs/data/ice/icd10_transcode.pdf [cited 2023 May 20].

[21] Hu G, Mamady K. Impact of changes in specificity of data recording on cause-specific injury mortality in the United States, 1999-2010. BMC Public Health. 2014 Sep 27;14(1):1010. 10.1186/1471-2458-14-1010 25262245 PMC4246427

[22] Liu LQ, Wan X. [Progress in research on redistribution methods for garbage codes in causes of death data]. Zhonghua Liu Xing Bing Xue Za Zhi. 2022 May 10;43(5):784–8. Chinese. 35589588 10.3760/cma.j.cn112338-20211025-00818

[23] Age standardization of rates. A new WHO standard. Geneva: World Health Organization; 2001. Available from: https://seer.cancer.gov/stdpopulations/world.who.html [cited 2023 May 20].

[24] Human development report 2020. New York: United Nations Development Programme; 2020. Available from: https://hdr.undp.org/content/human-development-report-2020 [cited 2023 May 20].

[25] Mathers CD, Fat DM, Inoue M, Rao C, Lopez AD. Counting the dead and what they died from: an assessment of the global status of cause of death data. Bull World Health Organ. 2005 Mar;83(3):171–7. 15798840 PMC2624200

[26] Bhatta S, Mytton J, Joshi E, Bhatta S, Adhikari D, Manandhar SR, et al. Development and evaluation of a community surveillance method for estimating deaths due to injuries in rural Nepal. Int J Environ Res Public Health. 2021 Aug 24;18(17):8912. 10.3390/ijerph18178912 34501502 PMC8430737

[27] Zarocostas J. Libya: war and migration strain a broken health system. Lancet. 2018 Mar 3;391(10123):824–5. 10.1016/S0140-6736(18)30505-1 29508734

[28] Douedari Y, Howard N. Perspectives on rebuilding health system governance in opposition-controlled Syria: a qualitative study. Int J Health Policy Manag. 2019 Apr 1;8(4):233–44. 10.15171/ijhpm.2018.132 31050968 PMC6499905

[29] WHO mortality database. Geneva: World Health Organization; 2020. Available from: https://www.who.int/news-room/questions-and-answers/item/who-mortality-database [cited 2023 May 20].

[30] Lorkowski J, Jugowicz A. Shortage of physicians: a critical review. Adv Exp Med Biol. 2021;1324:57-62. 10.1007/5584_2020_601 33346901

[31] Leber AL, Peterson E, Dien Bard J. Personnel Standards and Workforce Subcommittee, American Society for Microbiology. the hidden crisis in the times of COVID-19: critical shortages of medical laboratory professionals in clinical microbiology. J Clin Microbiol. 2022 Aug 17;60(8):e0024122. 10.1128/jcm.00241-22 35658527 PMC9383190

[32] Geneviève LD, Martani A, Mallet MC, Wangmo T, Elger BS. Factors influencing harmonized health data collection, sharing and linkage in Denmark and Switzerland: A systematic review. PLoS One. 2019 Dec 12;14(12):e0226015. 10.1371/journal.pone.0226015 31830124 PMC6907832

[33] Zuiderwijk A, Janssen M. 2014. The negative effects of open government data - investigating the dark side of open data. In: Proceedings of the 15th Annual International Conference on Digital Government Research. dg.o '14; 2014 Jun 18–21; Aguascalientes, Mexico. New York: Association for Computing Machinery; 2014. pp. 147–52. 10.1145/2612733.2612761 10.1145/2612733.2612761

[34] Shahraz S, Bhalla K, Lozano R, Bartels D, Murray CJ. Improving the quality of road injury statistics by using regression models to redistribute ill-defined events. Inj Prev. 2013 Feb;19(1):1–5. 10.1136/injuryprev-2011-040178 22505634

[35] Lee YR, Kim YA, Park SY, Oh CM, Kim YE, Oh IH. Application of a modified garbage code algorithm to estimate cause-specific mortality and years of life lost in Korea. J Korean Med Sci. 2016 Nov;31 Suppl 2(Suppl 2):S121-8. 10.3346/jkms.2016.31.S2.S121 27775249 PMC5081293

[36] Ning P, Schwebel DC, Chu H, Zhu M, Hu G. Changes in reporting for unintentional injury deaths, United States of America. Bull World Health Organ. 2019 Mar 1;97(3):190–9. 10.2471/BLT.18.215327 30992632 PMC6453323

[37] Nyondo T, Msigwa G, Cobos D, Kabadi G, Macha T, Karugendo E, et al. Improving quality of medical certification of causes of death in health facilities in Tanzania 2014-2019. BMC Health Serv Res. 2021 Sep 13;21(S1) Suppl 1:214. 10.1186/s12913-021-06189-7 34511104 PMC8436444

[38] Franca EB, Ishitani LH, Teixeira RA, Cunha CCD, Marinho MF. Improving the usefulness of mortality data: reclassification of ill-defined causes based on medical records and home interviews in Brazil. Rev Bras Epidemiol. 2019 Nov 28;22 Suppl 3(Suppl 3):e190010.supl.3. . 10.1590/1980-549720190010.supl.3 31800849

[39] WHO civil registration and vital statistics strategic implementation plan 2021–2025. Geneva: World Health Organization; 2021. Available from: https://apps.who.int/iris/bitstream/handle/10665/342847/9789240022492-eng.pdf?sequence=1&isAllowed=y [cited 2023 May 20].

[40] SCORE for health data technical package [internet]. Geneva: World Health Organization; 2021. Available from: https://www.who.int/data/data-collection-tools/score [cited 2023 May 20].

[41] Hart JD, Sorchik R, Bo KS, Chowdhury HR, Gamage S, Joshi R, et al. Improving medical certification of cause of death: effective strategies and approaches based on experiences from the Data for Health Initiative. BMC Med. 2020 Mar 9;18(1):74. 10.1186/s12916-020-01519-8 32146900 PMC7061467

[42] Mikkelsen L, Moesgaard K, Hegnauer M, Lopez AD. ANACONDA: a new tool to improve mortality and cause of death data. BMC Med. 2020 Mar 9;18(1):61. 10.1186/s12916-020-01521-0 32146907 PMC7061487

[43] Dong H, Falis M, Whiteley W, Alex B, Matterson J, Ji S, et al. Automated clinical coding: what, why, and where we are? NPJ Digit Med. 2022 Oct 22;5(1):159. 10.1038/s41746-022-00705-7 36273236 PMC9588058

[44] Chen Y, Chen H, Lu X, Duan H, He S, An J. Automatic ICD-10 coding: deep semantic matching based on analogical reasoning. Heliyon. 2023 Apr 19;9(4):e15570. 10.1016/j.heliyon.2023.e15570 37151662 PMC10161690

[45] Chen PF, He TL, Lin SC, Chu YC, Kuo CT, Lai F, et al. Training a deep contextualized language model for International Classification of Diseases, 10th Revision Classification via federated learning: model development and validation study. JMIR Med Inform. 2022 Nov 10;10(11):e41342. 10.2196/41342 36355417 PMC9693720

